# Supplementary material for: Semisynthetic fluorescent pH sensors for imaging exocytosis and endocytosis
Source: Nat Commun. 2017 Nov 10;8:1412. doi: 10.1038/s41467-017-01752-5 (PMC5680258; doi:10.1038/s41467-017-01752-5)
Supplement: Supplementary file 1 — Supplementary Info [file 41467_2017_1752_MOESM1_ESM.docx]

**
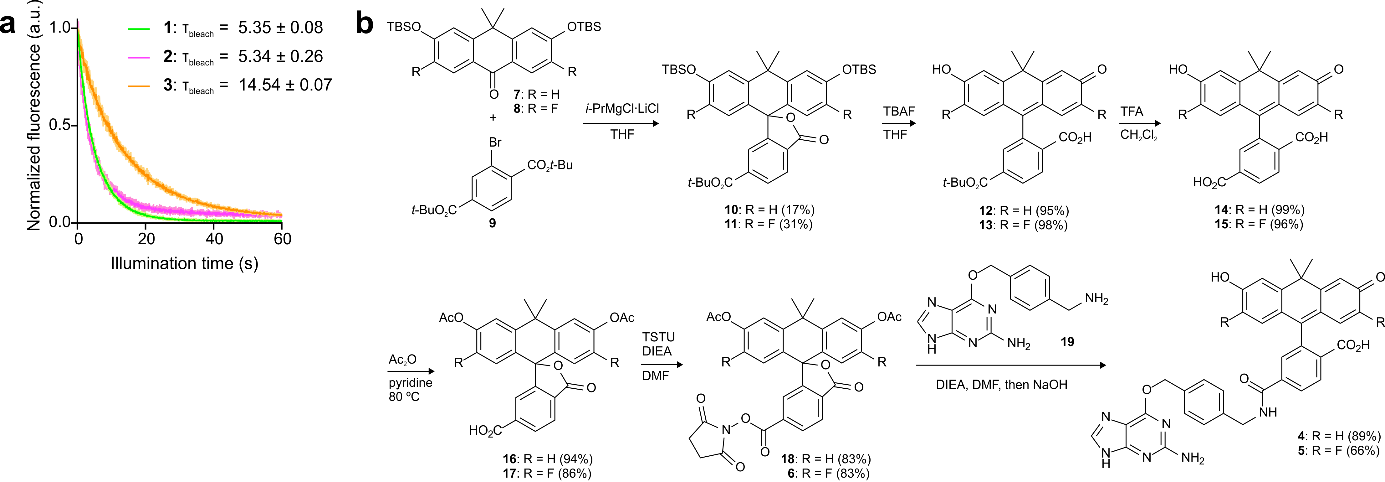
**

**Supplementary Figure 1. Photobleaching rates and synthesis steps of CFl, VO and derivatives.** This supplementary figure is related to Figure 1. **a**, Photobleaching rates of fluorescein (**1,** green), carbofluorescein (**2,** pink), or Virginia Orange (**3,** orange) at 1 µM solutions in 50 mM sodium borate buffer, dispersed in microdrops in octanol. **b**, Synthesis steps of CFl and VO derivatives. See Methods for details.


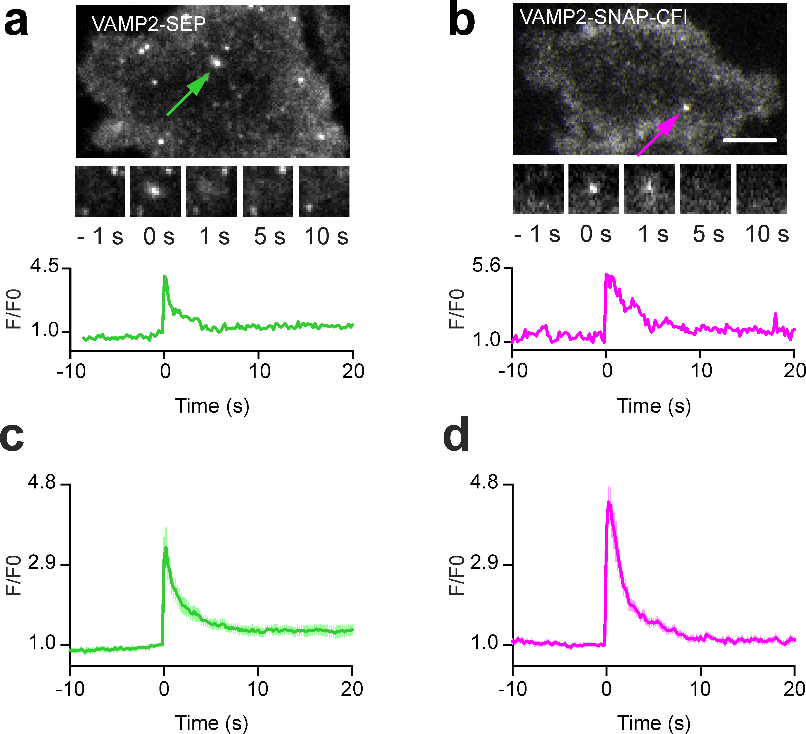


**Supplementary Figure 2. Detection of exocytosis events in PC12 cells with VAMP2 fusion proteins.** This supplementary figure is related to Figure 2. (**a**) Fluorescence image of a PC12 cell expressing VAMP2-SEP with the inset indicated with the green arrow (top). Time-lapse and fluorescence of the exocytosis event (bottom), 3.5 × 3.5 μm. (**b**) Same as (**a**) for a PC12 cell expressing VAMP2-SNAP incubated with CFL-SNAP-tag ligand, scale bar: 5 μm. (**c**,**d**) Average relative fluorescence of events recorded in cells expressing VAMP2-SEP (**d**, 34 events in 3 cells) or VAMP2-SNAP-CFl (**e**, 37 events in 5 cells). Error bars show ± s.e.m.


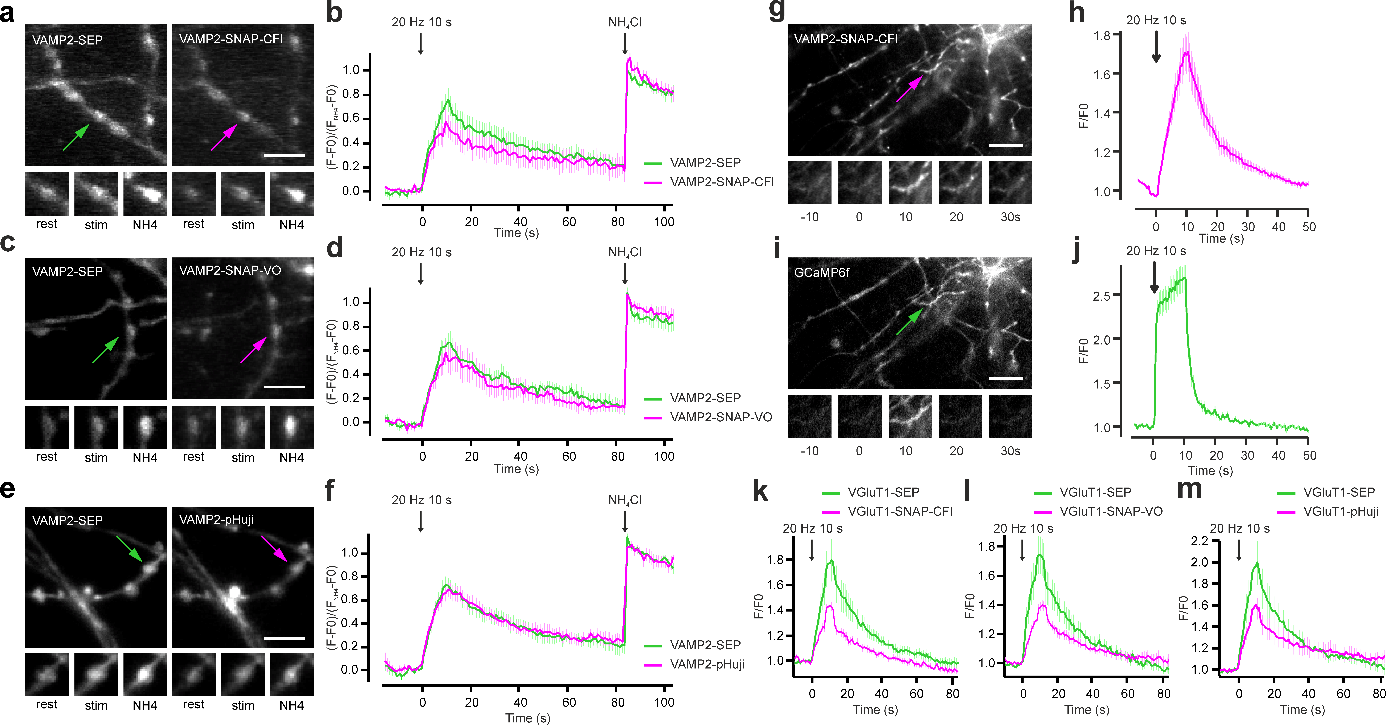


**Supplementary Figure 3. Detection of synaptic vesicle exocytosis and recycling in hippocampal neurons.** This supplementary figure is related to Figure 3. (**a**-**f**) Normalization of the response amplitude to the total unquenched fluorescence. (**a**) Fluorescence images of hippocampal axons expressing VAMP2-SEP and VAMP2-SNAP-CFl with the locations of vesicle fusion indicated with arrows (top); scale bar: 5 µm. Time-lapse images of the exocytosis events (bottom). (**b**) Average VAMP2-SEP (green) and VAMP2-SNAP-CFl (magenta) fluorescence signals in response to field stimulation for 10 s at 20 Hz and after application of 50 mM NH_4_Cl (n = 6). (**c**) Fluorescence images of hippocampal axons expressing VAMP2-SEP (left) and VAMP2-SNAP-VO (right) with the insets indicated with arrows (top). Time-lapse of the exocytosis events (bottom). Scale bar: 5 µm. (**d**) Average VAMP2-SEP (green) and VAMP2-SNAP-VO (magenta) fluorescence signals in response to field stimulation for 10 s at 20 Hz and after application of 50 mM NH_4_Cl (n = 7). (**e**) Fluorescence images of hippocampal axons expressing VAMP2-SEP (left) and VAMP2-pHuji (right) with the insets indicated with arrows (top). Time-lapse of the exocytosis events (bottom). Scale bar: 5 µm. (**f**) Average VAMP2-SEP (green) and VAMP2-pHuji (magenta) fluorescence signals in response to field stimulation for 10 s at 20 Hz and after application of 50 mM NH_4_Cl (n = 8). (**g-j**) Simultaneous detection of synaptic vesicle cycle and calcium transients in hippocampal neurons. (**g,i**) Fluorescence images of hippocampal axons expressing VAMP2-SNAP-CFl (**g**) and GCaMP6f (**i**). Scale bar: 10 µm. (**h,j**) Average VAMP2-SNAP-CFl (**h**) and GCaMP6f (**j**) fluorescence signals in the same boutons in response to field stimulation for 10 s at 20 Hz. n = 26. Data are represented as mean ± s.e.m. (**k**-**m**) Detection of exocytosis events in hippocampal neurons with VGluT1 fusion proteins. Average VGluT1-SEP (green) and VGluT1-SNAP-CFl (**k**, magenta, n = 37), VGluT1-SNAP-VO (**l**, magenta, n = 35) or VGluT1-pHuji (**m**, magenta, n = 26) fluorescence signals in response to field stimulation for 10 s at 20 Hz. Error bars show ± s.e.m.

**Supplementary Figure 4.** ^1^H NMR spectrum of **10**

**Supplementary Figure 5.** ^13^C NMR spectrum of **10**

**Supplementary Figure 6.** ^1^H NMR spectrum of **11**

**Supplementary Figure 7.** ^13^C NMR spectrum of **11**

**Supplementary Figure 8.** ^1^H NMR spectrum of **12**

**Supplementary Figure 9** ^13^C NMR spectrum of **12**

**Supplementary Figure 10.** ^1^H NMR spectrum of **13**

**Supplementary Figure 11** ^13^C NMR spectrum of **13**

**Supplementary Figure 12.** ^1^H NMR spectrum of **14**

**Supplementary Figure 13.** ^13^C NMR spectrum of **14**

**Supplementary Figure 14.** ^1^H NMR spectrum of **15**

**Supplementary Figure 15.** ^13^C NMR spectrum of **15**

**Supplementary Figure 16.** ^1^H NMR spectrum of **16**

**Supplementary Figure 17.** ^13^C NMR spectrum of **16**

**Supplementary Figure 18.** ^1^H NMR spectrum of **17**

**Supplementary Figure 19.** ^13^C NMR spectrum of **17**

**Supplementary Figure 20.** ^1^H NMR spectrum of **18**

**Supplementary Figure 21.** ^13^C NMR spectrum of **18**

**Supplementary Figure 22.** ^1^H NMR spectrum of **6**

**Supplementary Figure 23.** ^13^C NMR spectrum of **6**

**Supplementary Figure 24.** ^1^H NMR spectrum of **4**

**Supplementary Figure 25.** ^13^C NMR spectrum of **4**

**Supplementary Figure 26.** ^1^H NMR spectrum of **5**


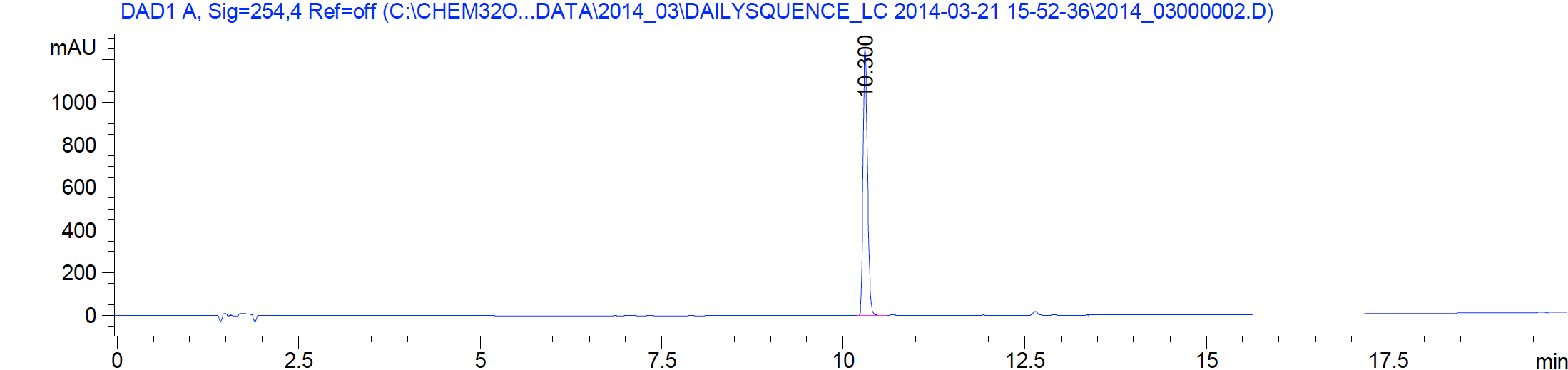


**a**

**b**


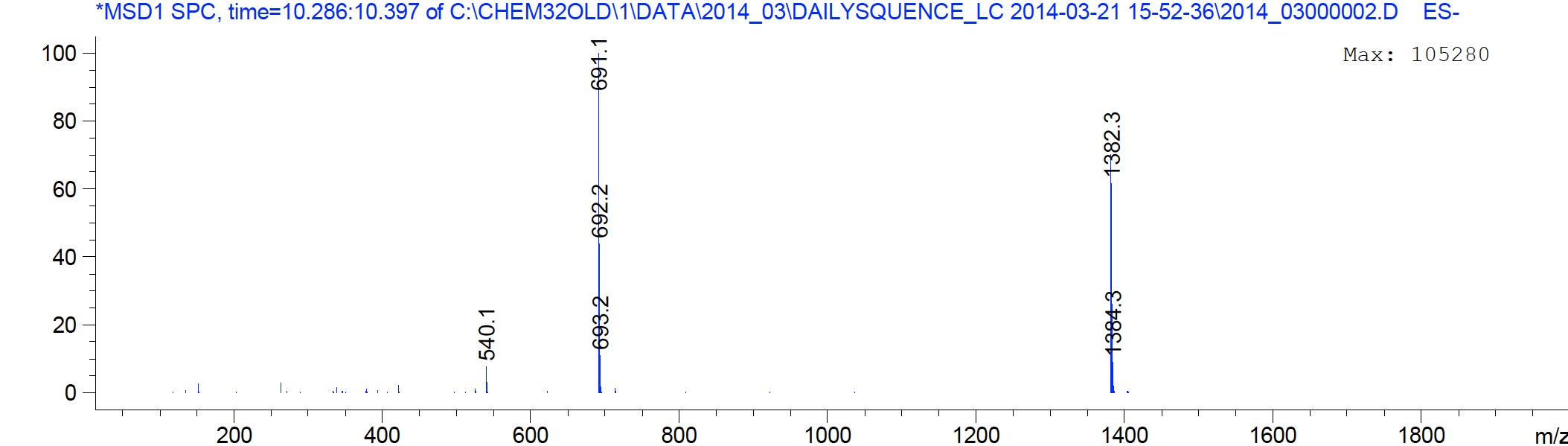


**Supplementary Figure 27:** Tandem liquid chromatography–mass spectrometry (LC-MS) data for **5**. (**a**) Chromatogram at absorbance = 254 nm; found 98.6% purity. (**b**) Mass spectrum of peak at *t* = 10.3 min; ESI positive mode; expected mass for C_37_H_29_F_2_N_6_O_6_ [M+H]^+^ = 691.2.

**Supplementary Table 1**: Properties of the red, pH-sensitive, fluorophores used in this study

| Fluorophore | Emission Peak  (nm) | Extinction coeff  (M^-1^cm^-1^) | Quantum Yield | Source |
| --- | --- | --- | --- | --- |
| pHuji | 598 | 31000 | 0.22 | Shen et al. 2014 |
| pHoran4 | 561 | 83000 | 0.66 | Shen et al. 2014 |
| Carbofluorescein | 567 | 108000 | 0.62 | Grimm et al. 2016 |
| Virginia Orange | 581 | 90900 | 0.40 | Grimm et al. 2016 |
